# Targeting the epichaperome as an effective precision medicine approach in a novel *PML*-*SYK* fusion acute myeloid leukemia

**DOI:** 10.1038/s41698-021-00183-2

**Published:** 2021-05-26

**Authors:** Mayumi Sugita, David C. Wilkes, Rohan Bareja, Kenneth W. Eng, Sarah Nataraj, Reyna A. Jimenez-Flores, LunBiao Yan, Jeanne Pauline De Leon, Jaclyn A. Croyle, Justin Kaner, Swathi Merugu, Sahil Sharma, Theresa Y. MacDonald, Zohal Noorzad, Palak Panchal, Danielle Pancirer, Shuhua Cheng, Jenny Z. Xiang, Luke Olson, Koen Van Besien, David S. Rickman, Susan Mathew, Wayne Tam, Mark A. Rubin, Himisha Beltran, Andrea Sboner, Duane C. Hassane, Gabriela Chiosis, Olivier Elemento, Gail J. Roboz, Juan Miguel Mosquera, Monica L. Guzman

**Affiliations:** 1 Department of Medicine, Division of Hematology/Oncology, Weill Cornell Medicine, New York, NY USA; 2 Caryl and Israel Englander Institute for Precision Medicine, Weill Cornell Medicine and NewYork Presbyterian, New York, NY USA; 3 Department of Pathology and Laboratory Medicine, Weill Cornell Medicine, New York, NY USA; 4 Department of Physiology and Biophysics, Weill Cornell Medicine, New York, NY USA; 5 Institute for Computational Biomedicine, Weill Cornell Medicine, New York, NY USA; 6 Chemical Biology Program, Memorial Sloan Kettering Cancer Center, New York, NY USA; 7 Department of Pharmacology, Weill Cornell Medicine, New York, NY USA; 8 Present Address: Bern Center of Precision Medicine, Universität of Bern, Bern, Switzerland; 9 Present Address: Division of Medical Oncology, Dana Farber Cancer Institute, Boston, MA USA

**Keywords:** Acute myeloid leukaemia, Targeted therapies

## Abstract

The epichaperome is a new cancer target composed of hyperconnected networks of chaperome members that facilitate cell survival. Cancers with an altered chaperone configuration may be susceptible to epichaperome inhibitors. We developed a flow cytometry-based assay for evaluation and monitoring of epichaperome abundance at the single cell level, with the goal of prospectively identifying patients likely to respond to epichaperome inhibitors, to measure target engagement, and dependency during treatment. As proof of principle, we describe a patient with an unclassified myeloproliferative neoplasm harboring a novel *PML-SYK* fusion, who progressed to acute myeloid leukemia despite chemotherapy and allogeneic stem cell transplant. The leukemia was identified as having high epichaperome abundance. We obtained compassionate access to an investigational epichaperome inhibitor, PU-H71. After 16 doses, the patient achieved durable complete remission. These encouraging results suggest that further investigation of epichaperome inhibitors in patients with abundant baseline epichaperome levels is warranted.

## Introduction

Precision medicine (PM) approaches in cancer treatment are designed to offer patient-specific, mechanistically driven selection of therapeutic strategies. Development of such “bespoke” treatment is hoped to improve current outcomes with conventional chemotherapy, especially in diseases like acute myeloid leukemia (AML), in which outcomes are especially dismal. PM takes into consideration the unique tumor molecular landscape or patient’s signature to determine potential therapeutic interventions. At the Englander Institute for Precision Medicine (EIPM) of Weill Cornell Medicine (WCM) and New York‐Presbyterian Hospital (NYP), we have implemented a program that includes whole‐exome and RNA-sequencing to facilitate the determination of tumor molecular landscapes, and ex vivo drug screening to identify drug sensitivity and resistance^1–3^.

In addition to the genome, PM can be also implemented at the proteome level. Cellular functions are performed by sets of proteins organized into interconnected protein–protein interaction (PPI) networks. We recently reported that, independent of the tissue of origin and the genetic background of tumors, stressors associated with malignant transformation aberrantly rewire PPI networks to provide a survival advantage under conditions of stress. These proteome-wide changes are enabled by the restructuring of the chaperome, an assembly of molecular chaperones, co-chaperones and other co-factors, into new entities termed epichaperome networks^1,4^. Unlike the chaperome, these structures do not act in folding per se but rather as multimolecular scaffolds that pathologically remodel proteome-wide cellular processes^1,5^. By providing the backbone upon which protein networks become aberrantly reorganized, epichaperomes are essential to cancer cell viability. Therefore, they present vulnerability and in turn, a target for therapeutic intervention with drugs that impair epichaperome formation and/or its function.

There is a positive correlation between epichaperome formation and vulnerability of tumors to epichaperome inhibitors^1,4,6^, suggesting that epichaperome measurements, which represent a functional analysis for proteome vulnerability to epichaperome inhibitors of patients’ cells, may provide a rational basis for PM. We here report on the implementation and clinical success of our epichaperome-directed diagnostic assay in a patient with refractory AML, which tumor harbored a novel *PML-SYK* fusion and epichaperome-driven aberrant PPI networks.

## Results

### Clinical case presentation

**Fig. 1:**
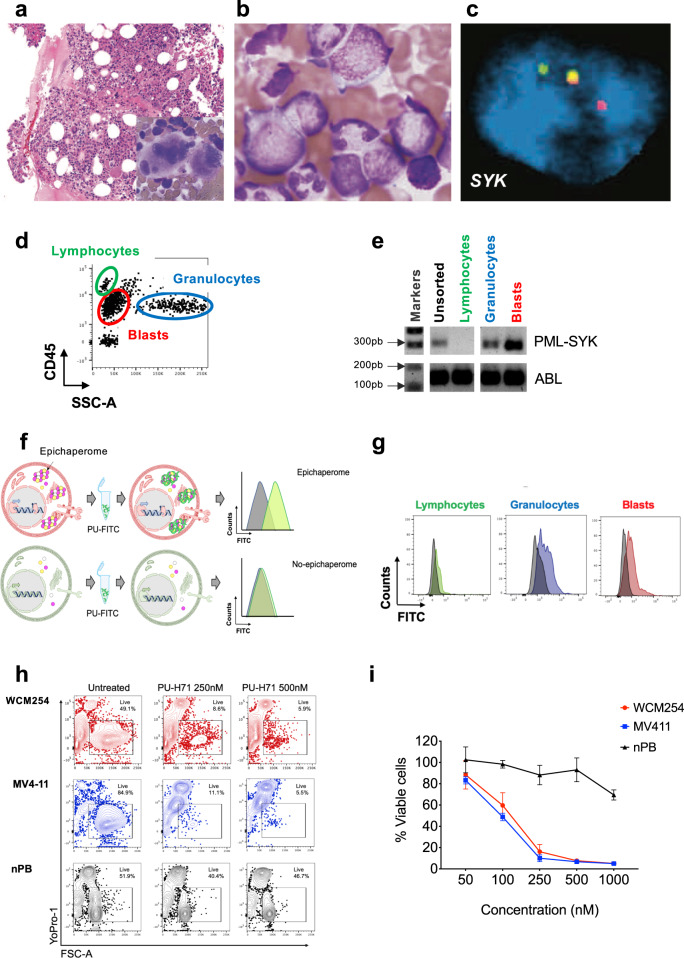
A case of relapsed/refractory AML (WCM254) with a novel *PML-SYK* fusion gene and transcript with epichaperome enrichment. **a** Hematoxylin and eosin (H&E) staining of the bone marrow (BM) biopsy showing hypercellular BM with unclassified myeloproliferative disorder at disease onset. Hematopathology evaluation from 45 months prior this study. **b** BM aspirate shows increased myeloblasts as patient progressed to acute myeloid leukemia (AML). Hematopathology evaluation 17 months prior to this study. **c** FISH analysis for *SYK* showing break apart (rearranged) green and red signals, and yellow (unrearranged) allele (for *PML* FISH data see Supplementary Fig. 1). **d** Representative gating of leukocyte subpopulations evaluated with flow cytometry. Blasts, lymphocytes and granulocytes were sorted by gating on SSC-A vs. CD45 from bone marrow (BM) sample at baseline. **e** Agarose gel electrophoresis of PCR amplicon showing positive *PML-SYK* fusion gene transcripts in bulk population and sorted populations indicated in **d**. **f** Schematic representation of cells with high (top row) or no epichaperome (bottom row) with corresponding expected flow histograms in PU-FITC biding assay. PU-FITC binds to intracellular epichaperome with high affinity and gives signal indicating abundance of epichaperome. **g** Preclinical evaluation of epichaperome abundance in lymphocytes (green), granulocytes (blue) and blasts (red) by performing PU-FITC-binding assay. The intensity of FITC fluorescence indicates the level of epichaperome and unstained control is shown in gray. **h** and **i** Viability assay with cells from WCM254 (red), MV4;11 (blue, control AML cell line with high epichaperome and with high sensitivity to PU-H71) or normal peripheral blood (nPB) mononuclear cells (black) treated with escalating doses of PU-H71 for 72 h in vitro. Representative flow plots (**h**) and graphic representation at increasing doses of PU-H71 (**i**). Experiments performed in triplicate, showing the mean and error bar represents the SD.

A 56-year-old woman with a history of prophylactic bilateral mastectomy for familial *BRCA* mutation, arthritis and leukocytoblastic vasculitis, and urticaria, had an incidental finding of leukocytosis in 2013. The bone marrow biopsy was hypercellular (>95%) with full myeloid maturation and 6% myeloblasts. Megakaryocytes were markedly increased, predominantly clustered in the spicules, and dysplastic (Fig. 1a). Cytogenetic analysis revealed t(9;15) involving the *PML* gene (Supplementary Fig. 1a, b). Molecular studies were negative for *BCR-ABL*, *PDGFRA/B, FGFR1* rearrangements, and *JAK2* mutation. The patient was diagnosed with an unclassified myeloproliferative disorder (MPD) and underwent matched unrelated donor (MUD) allogeneic stem cell transplantation conditioned with Fludarabine/Melphalan. The patient was in complete remission for a year but showed evidence of early relapse starting in 2014. She was treated with three cycles of azacitidine. The bone marrow biopsy demonstrated significant granulocytic hyperplasia, megakaryocytic atypia and increased CD34-positive cells with persistent t(9;15) involving *PML*. She had stable blood counts and a good performance status until approximately two years later, when she developed painful ulcerations of toes, thought to be an atypical presentation of sclerodermoid graft versus host disease (GVHD) complicated by underlying Raynaud’s disease and treated successfully with ruxolitinib. During this period of time, she had bone marrow and skin evidence of recurrent disease, along with significant splenomegaly. Neutrophils and platelets were preserved, she did not require transfusions, and she had no bleeding or infections. She was maintained on hydroxyurea, prednisone and ruxolitinib, but a few months later progressed to acute myeloid leukemia (AML) with fibrosis (Fig. 1b). The patient developed progressive splenomegaly and weight loss and was treated with two cycles of decitabine without response. At that point the patient was referred to the Englander Institute for Precision Medicine (EIPM)^7^ for evaluation in search for potential alternative and innovative treatment options. The EIPM’s ongoing efforts to identify tailored treatment options include a next-generation sequencing (NGS)-based clinical study, onto which the patient was enrolled (index case WCM254). The study had enrolled seven patients with myeloid neoplasia at the time (Supplementary Table 1), but prioritized additional testing and functional validation assays on our index case (WCM254) given her rapid progression to a poor prognosis AML.

### Identification of a novel *PML-SYK* gene fusion in myeloid neoplasia

Cytogenetic and break-apart FISH analyses of the patient showed t(9;15) (Supplementary Fig. 1a, b). Whole-exome sequencing with a control buccal swab and RNA-seq were performed. Mutations in *ETV6* and multiple *ASXL1* subclones were present, but no somatic alterations in actionable genes were found (Supplementary Table 1). However, a novel *PML-SYK* fusion, was detected from transcriptomic analysis via RNA-seq (Supplementary Fig. 1c–f). This fusion, with strong support of spanning pairs, was found using three different algorithms (see Methods). In the predicted putative protein resulting from the fusion, 417 AA are retained in PML and 331 AA are retained in SYK. The Zinc finger domain in PML is retained and the DUF3583 domain is broken. The tyrosine-protein kinase catalytic domain is retained in SYK. Furthermore, there was overexpression of *SYK* when RNA-seq data from the index case (WCM254) was compared with gene expression of the entire cohort at EIPM (Supplementary Fig. 1g). The novel *PML-SYK* gene fusion was validated by fluorescent in situ hybridization (FISH) (Fig. 1c). Blasts, lymphocytes and granulocytes were sorted by gating on SSC-A vs. CD45 from bone marrow (BM). Reverse-transcriptase polymerase chain reaction (RT-PCR) was used to evaluate RNA transcripts of *PML-SYK* gene fusion, which was detected in both blasts and granulocytes, but not the lymphocytes, suggesting that they are non-transformed (Fig. 1d, e). We confirmed that the lymphocytes are donor-derived from the male matched unrelated donor (MUD) transplant she previously received (Supplementary Fig. 1h).

### High epichaperome levels detected in *PML-SYK* fusion-positive cells

We have previously reported a positive correlation between the epichaperome and a hyperactivated signalosome in AML cells^6^, which was confirmed in this patient while evaluating the novel *PML-SYK* fusion. The malignant cells, but not the donor-derived lymphocytes, showed aberrantly activated signaling pathways, as evidenced by elevated levels of phospho-SYK (Y525/526), phospho-STAT5 (Y694) and phospho-ERK1/2 (T202/Y204) and phospho-S6 (S235/S236), measured by flow (Supplementary Fig. 2), suggesting that the epichaperome levels were likely to be elevated.

We next investigated the functional signature of the patient’s cells using our flow cytometry-based PU-FITC-binding assay (Fig. 1f, g), which measures epichaperome abundance at the single cell level^8^. Blasts and granulocytes bearing *PML-SYK* showed high epichaperome abundance, in contrast to the normal lymphocytes derived from the male matched unrelated donor (Fig. 1g).

### High epichaperome levels render cells sensitive to PU-H71

As epichaperome positivity is associated with sensitivity to epichaperome inhibition^1,9^, we investigated the ex vivo susceptibility of the patient’s cells to PU-H71, an investigational epichaperome inhibitor undergoing clinical evaluation in different types of cancers^9,10^. We treated cells with PU-H71 at different doses and evaluated cell viability by flow cytometry after 72 h of exposure. We found that the patient’s blast cells were as sensitive to PU-H71 as MV4-11 cells, which we previously reported to be epichaperome high and dependent on the epichaperome for survival^1,6^ (Fig. 1h, i, primary sample shown in red and MV-411 shown in blue). Importantly, healthy PB MNCs are not affected by PU-H71 treatment (Fig. 1h, i, shown in black). Colony-forming capacity was also impaired in cells obtained from PB baseline samples when exposed in vitro to PU-H71, suggesting a concomitant impact on stem/progenitor cells (Supplementary Fig. 3a, b).

### PU-H71 single-patient trial

**Fig. 2:**
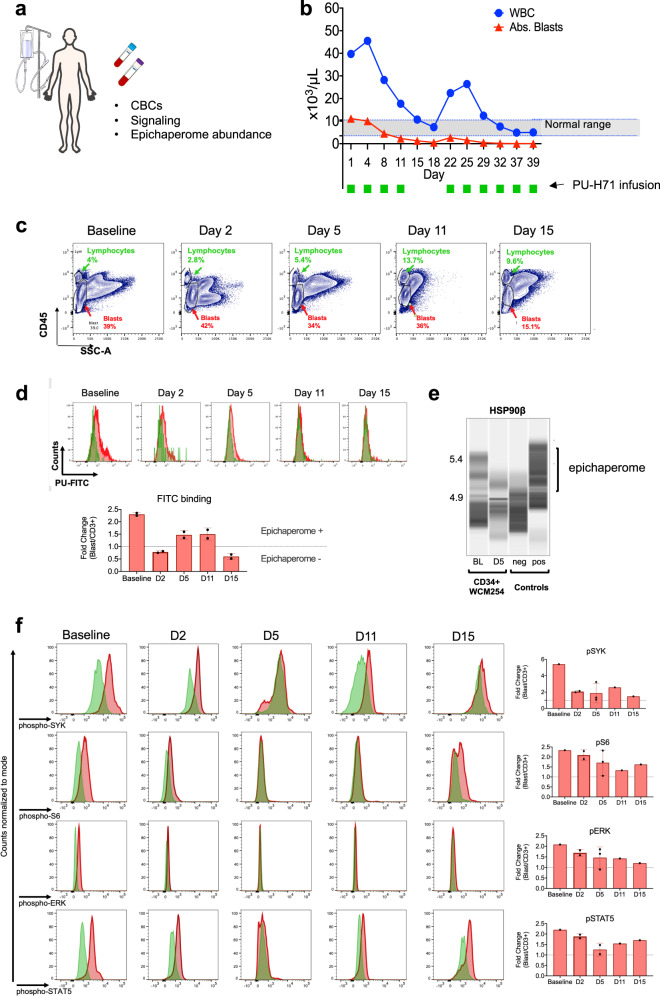
PU-H71 productively engages the epichaperome during treatment of patient WCM254. **a** During treatment with PU-H71, the patient WCM254 was monitored for complete blood counts (CBCs), signaling in leukocytes subsets, and epichaperome abundance in cells from peripheral blood and bone marrow. **b** The clinical course of patient WCM254 during the first ten doses of PU-H71. Absolute leukocyte (blue circle) and blast counts (red triangle) are indicated on each day they were assessed. Days on which the patient received a dose of PU-H71 are indicated with green squares. Gray shaded area indicates the normal range of leukocyte counts. **c** Chronological changes of percentages of lymphocytes (green) and blasts (red) in mononuclear cells at baseline and on day 2, 5, 11, and 15. Lymphocytes and blasts were gated on SSC-A vs. CD45. **d**, Epichaperome abundance determined by flow cytometry on day 0, 5, 11, and 15 is shown as a biomarker of response to PU-H71. Graph, mean ± SD of MFI of blasts (i.e., epichaperome +ve)/MFI of CD3 + lymphocytes (i.e., epichaperome -ve). **e** Isoelectric focusing shows long-lived multimeric HSP90 species (a biochemical signature of epichaperomes) in sorted CD34 + baseline (BL) blasts that decrease by day 5 of treatment (D5). Negative control, ASPC1 (epichaperome + ) homogenate; positive control, MDA-MB-468 homogenate (epichaperome-)^1^. **f** Cell subsets as in **c** and **d** were evaluated by flow cytometry for levels of intracellular phospho-Syk(Y525/526), phospho-STAT5(Y694), phospho-S6 (S235/S236) and phospho-ERK(T202/Y204). Graph, mean ± SD of MFI of blasts/MFI of CD3 + lymphocytes.

Based on the poor prognosis, lack of effective or alternative therapies, and laboratory findings demonstrating high epichaperome levels that predicted an increased likelihood of response to PU-H71, the patient was granted compassionate access to this medication by the FDA. PU-H71 was administered at 300 mg/m^2^, a dose reported to maximally engage the target in epichaperome-positive tumors^9^. She continued concomitant treatment with ruxolitinib, which was needed to control the sclerodermoid GVHD in her toes and had been shown to be safe in combination with PU-H71 in a previous clinical trial^9^. With the patient’s informed consent, we collected peripheral blood and bone marrow samples during the course of treatment for evaluation of epichaperome abundance and cell signaling (Fig. 2a).

**Fig. 3:**
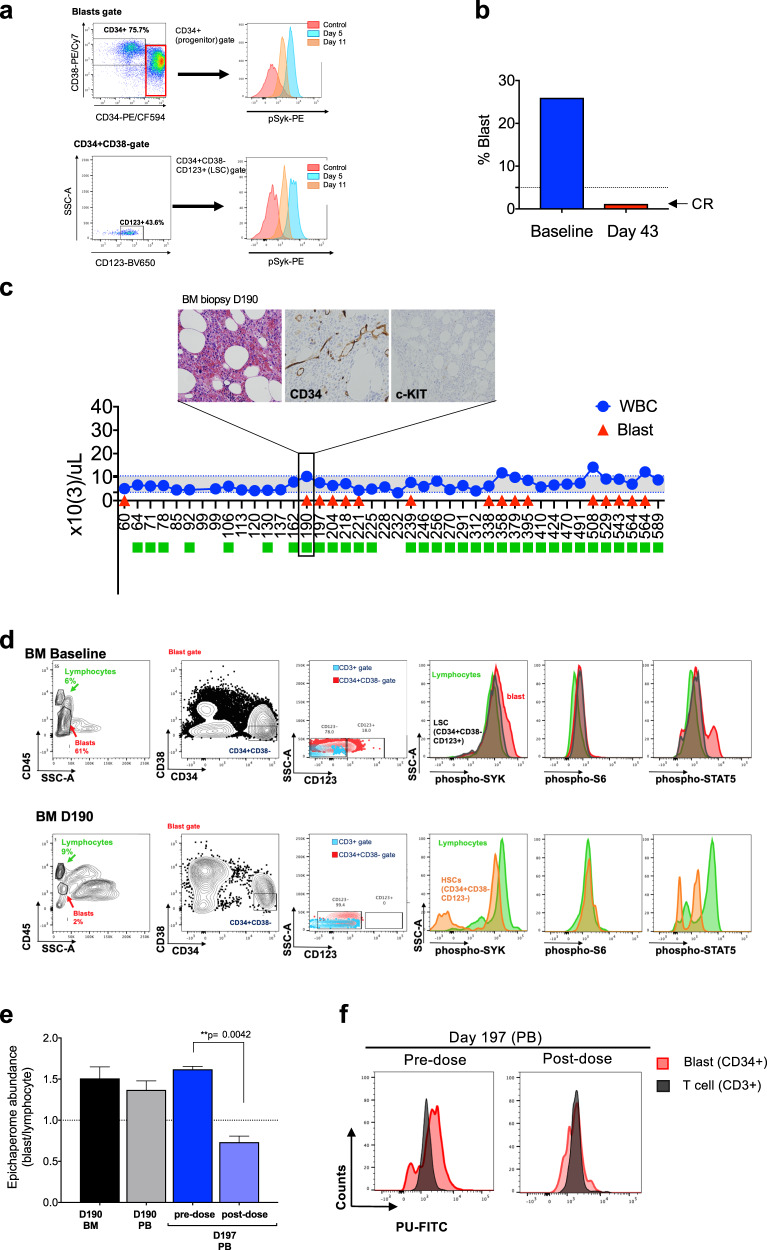
Long-term evaluation of disease and the impact of treatment in stem cell and progenitor compartments during administration of PU-H71. **a** Evaluation of activated intracellular signaling in AML CD34 + progenitor cells (top) and CD34 + CD38-CD123 + leukemic stem cells (bottom) at day 5 and 11 during PU-H71 therapy. Red shows florescence minus one (FMO) control. **b** Clinical assessment of the percentage of blasts in bone marrow at baseline and on day 43 after 10 does of PU-H71. Bone marrow aspirate showed <5% blasts with confirming complete remission (CR). **c** The clinical course of the patient WCM254 from day 60 to day 589. Blue circles, absolute leukocyte counts; red triangles blast counts in PB. PU-H71 administration, green square. Gray shaded area, normal range of leukocyte counts. The patient remains in clinical remission under continuous treatment of PU-H71 for over 600 days. (Inset) Pathological evaluation of bone marrow biopsy at day 190. H&E staining of bone marrow shows slightly hypercellular marrow (left panel) but without evidence of myeloblasts by cytomorphology and immunohistochemistry for CD34 and c-KIT (center and right panels). **d** Evaluation of intracellular signaling in leukemic stem cells (LSCs, CD34 + CD38-CD123 + ) and hematopoietic stem cells (HSCs, CD34 + CD38-CD123-) in bone marrow aspirates at baseline (top row) and on day 190 (bottom row). LSC population (CD34 + CD38-CD123 + ) at baseline showing hyperactivated signaling was replaced with CD34 + CD38-CD123- hematopoietic stem cells (HSCs) populations with downregulated signaling on day 190. **e** Epichaperome abundance evaluated by flow cytometry on day 190 and day 197. Epichaperome increase in cells from both BM and PB was detected on day 190. Rapid and significant decrease of epichaperome abundance on day 197 after one dose of PU-H71 suggested target engagement. **p = 0.0042, one-way ANOVA. **f** Flow cytometry histogram of epichaperome abundance in CD34 + blasts (red) and T-lymphocytes (gray) at pre- and at 4 h post-infusion on day 197 showed rapid decrease of epichaperome abundance in CD34 + blasts after PU-H71 administration.

PU-H71 was administered initially twice per week for 2 weeks. We observed an immediate decrease in the number of blasts present in the peripheral blood and a decrease in the total white blood cell count to normal levels (Fig. 2b). We also confirmed the decrease in blasts by flow cytometry in ficolled PB samples (Fig. 2c). The decline in blasts and normalization of white blood cell counts were paralleled by decreased epichaperome (Fig. 2d). Epichaperome abundance was monitored using flow cytometry, and levels were evaluated in PB samples obtained prior to infusion of the drug. We also confirmed epichaperome decrease in sort-purified blasts obtained at both baseline and day 5 using an alternative method, i.e., a capillary-based immunoassay platform that combines isoelectric focusing (IEF) with immunoblotting^1^ (Fig. 2e). Upon epichaperome binding, PU-H71 disassembles epichaperomes into individual, normal, folding chaperones^9,11^, indicating that the epichaperome decrease we measure early in the treatment regimen reflects productive target engagement by the 300 mg/m^2^ administered dose of PU-H71. Confirming target engagement by PU-H71, we observed that the reduction in peripheral blood blast levels was paralleled by a rebalance in the activity of PPI networks, as evidenced by a decrease in the activity of signaling pathways (see levels of phospho-SYK (Y525/526), phospho-STAT5 (Y694), and phospho-S6 (S235/S236) in the bottom panels of Fig. 2f), Furthermore, PU-H71 treatment also rebalanced the activity of PPI networks in AML progenitor (CD34 + ) and stem cell populations (CD34 + CD38-CD123 + ) (Fig. 3a).

When treatment was interrupted during the third week, the blasts and WBC counts increased, followed again by immediate response when PU-H71 was restarted on day 22 (Fig. 2b). Importantly, by day 43, after 10 doses of PU-H71, the bone marrow showed <5% blasts (Fig. 3b). At that time, the patient’s splenomegaly and constitutional symptoms had also completely resolved. After 16 doses over 3 months, the patient attained complete remission (CR), with normalization of peripheral blood counts. Colony-forming assays also showed that cells obtained from the BM were able to form erythroid colonies, which were not observed when assessing the baseline BM sample (Supplementary Fig. 4). At this point, we adopted a maintenance regimen based on laboratory correlative studies, with PU-H71 administered weekly between days 64–78, then every two weeks between days 92–106, and then monthly until day 190.

Blasts in peripheral blood and bone marrow remained undetectable throughout the 162-day period (Fig. 3c). At day 190, the patient had 1% blasts in the peripheral blood, however, bone marrow aspirate was performed and showed no evidence of relapse (Fig. 3c, inset). Phenotypically defined leukemia stem cells (CD34 + CD38-CD123 + ) and the remaining stem/progenitor cells (CD34 + CD38-CD123-) showed no heightened activity of signaling networks (Fig. 3d). Epichaperome levels, however, were increasing at day 190, 28 days after the last dose of PU-H71. Based on these levels, we restarted weekly administration of PU-H71 and seven days later the epichaperome abundance, evaluated pre- and post-infusion, revealed significantly decreased epichaperome levels (Fig. 3e, f; *p* = 0.0042). After another 28 days, peripheral blasts were no longer detected in the blood. This data suggests that WCM254 cells are not acquiring resistance to the treatment.

**Fig. 4:**
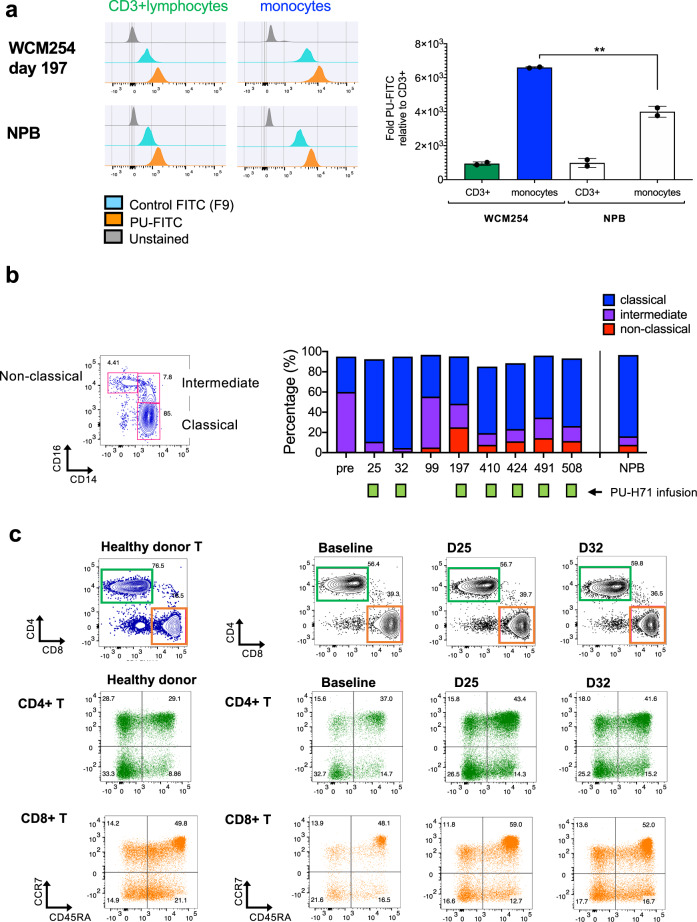
Monocyte population displays epichaperome abundance and immunophenotyped changes during the course of treatment with PU-H71 while T lymphocytes are not affected. **a** Epichaperome abundance in monocytes and CD3 + T-cell lymphocytes in WCM254 compared with normal PB lymphocytes (NPB). Graph, mean ± SD of MFI of monocytes/MFI of CD3 + lymphocytes. **b** Representative monocyte subsets (classical, intermediate, and non-classical) based on CD14 and CD16 expression in normal healthy donor (left). Chronological changes in monocyte subsets in patient WCM254 during PU-H71 therapy (right). Pro-inflammatory subsets CD14^high^CD16 + (intermediate) and CD14^low^CD16 + (non-classical) decreased after PU-H71 treatment reflecting proportions similar to normal monocyte subsets. **c** Phenotyping of T-lymphocytes in WCM254 at baseline, day 25 and day 32, with representative T cells from a healthy donor shown on the left. Proportion of CD4 + and CD8 + T lymphocytes and naive/central memory/effector/terminally differentiated effector T cells of each CD4 + and CD8 + T cells did not show changes in WCM254 under PU-H71 treatment (right three columns).

While monitoring epichaperome levels during treatment using multiparameter flow cytometry, we observed that in addition to blast cells, monocytes displayed epichaperome positivity (Fig. 4a) while T cells (donor-derived) remained epichaperome negative. Thus, we immunophenotyped monocyte and T-cell subsets, which include CD16 + CD14^low^ pro-inflammatory non-classical monocytes, CD16^neg^CD14^+^ classical monocytes, and CD16 + CD14 + intermediate monocytes^12,13^; CD4^+^ helper T cells and CD8^+^ cytotoxic T cells; as well as naive, effector memory, and memory T-cell subsets^14,15^ to determine whether PU-H71 treatment had an impact on the immune microenvironment. At baseline, we found an aberrant distribution in the proportions of CD16 + CD14^low^ pro-inflammatory non-classical monocytes^12^ and CD16 + CD14 + intermediate monocytes^12,13^, two populations that exhibit pro-inflammatory behavior and may affect T-cell function^14,15^. During PU-H71 treatment, we observed a rebalance in monocyte populations to proportions determined in a healthy donor (Fig. 4b). We did not find significant changes in the proportions of T-cell subsets, suggesting that PU-H71 does not directly affect T-cell subsets or have a negative effect on T-cell proportions, however were unable to assess T-cell function (Fig. 4c). Overall, the data suggest that PU-H71 could positively impact immune cells, a phenomenon that may contribute to its long-term anti-leukemia response; this potential mechanism is currently under further investigation.

The patient has continued treatment with PU-H71 every 2–3 weeks for >600 days. As of this writing, she is clinically well and without any grade 2–4 drug-related toxicity. She has noted intermittent grade 1 myalgias and joint swelling, as well as fatigue for 1–2 days following PU-H71 infusions. We are continuing active surveillance of peripheral blood epichaperome levels and immune profiles.

## Discussion

The epichaperome is a clinical biomarker that indicates potential tumor vulnerability to epichaperome inhibitors, such as PU-H71. Preclinical studies of PU-H71 have shown high cytotoxicity against cancer cell lines of several tumor types and potent, lasting growth inhibitory effects in mouse xenografts^4^. Based on these results, a first in-human trial of PU-H71 was initiated in order to establish safety and tolerability, and to characterize its pharmacokinetic profile in patients with advanced solid tumors refractory to standard treatment. No dose limiting toxicities occurred^16^, but clinical efficacy in the unselected clinical trial population was limited. Our PU-FITC-binding assay for epichaperome levels at the single cell level offers a unique opportunity to identify patients with increased likelihood of response to PU-H71, as illustrated herein with case WCM254. The remarkable clinical success of PU-H71 in this poor prognosis AML patient has potentially broad implications. The PU-FITC flow cytometry-based assay is a feasible, low-cost screening tool that can accurately interrogate tumors for epichaperome abundance. The assay has been tested in cell lines^1^, solid tumor organoids (unpublished data) and primary AML samples (^1^and current study). In our patient, PU-H71 was also shown to affect monocytes, and we speculate that PU-H71 may have decreased the presence of an immune-suppressive microenvironment, thus allowing donor-derived T-lymphocytes to exert immune surveillance. Thus, studies of the potential role of PU-H71 in the regulation of the immune microenvironment and the implications for post-transplant patients with relapsed AML are warranted, as these patients have limited clinical options and poor outcomes. This report illustrates the power of precision medicine approaches beyond panel sequencing in tailoring effective personalized treatment approaches. Our results demonstrate a successful clinical application of the PU-FITC-binding assay in a post-transplant, relapsed AML patient with *PML-SYK* fusion whose tumor cells demonstrated epichaperome abundance. We believe that incorporating the epichaperome as a biomarker will facilitate identification of cancer patients who may benefit from treatment with epichaperome inhibitors.

## Methods

### Cells and cell culture

Patient samples were acquired with Weill Cornell Medicine Institutional Review Board (IRB) approval. De-identified healthy donor blood samples were obtained from the New York Blood Center with the approval by NYBC IRB. MV4;11 cell line was obtained from the ATCC (Manassas, VA, USA) and was authenticated and tested for mycoplasma. Primary samples were cultured in serum-free culture medium with cytokines (rh FLT3L, rh SCF, rh IL-3 and rh IL6)^6^. MV4;11 cells were cultured in Iscove’s Modified Dulbecco’s Medium (IMDM; ThermoFisher) supplemented with 10% fetal bovine serum (FBS) and 1% penicillin/streptomycin (ThermoFisher).

### Analysis of RNA-sequencing data

All reads were independently aligned with STAR_2.4.0f for sequence alignment against the human genome build hg19, downloaded via the UCSC genome browser [http://hgdownload.soe.ucsc.edu/goldenPath/hg19/bigZips/]x, and SAMTOOLS v0.1.19 for sorting and indexing reads^17,18^. Cufflinks (2.0.2) was used to evaluate expression values (FPKMS), and Gencode v19 GTF file for annotation^19,20^. FPKMs are transformed into *z*-scores by comparison with a baseline distribution using all samples from the IPM cohort. For fusion analysis, we used STAR-fusion (STAR-Fusion_v0.5.1), Fusioncatcher (v0.99.3e) and FusionSeq^21–23^) Fusions with significant support of junction reads and spanning pairs are then selected and manually reviewed. We also used oncofuse to estimate the oncogenic potential of fusions by looking at the protein domains broken and retained^24^.

### Fluorescence in situ hybridization

Fluorescence in situ hybridization (FISH) was performed using the LSI PML-RARA dual color dual fusion rearrangement probe (Vysis/Abbott Molecular Inc., Des Plains, IL) to determine the involvement of PML gene in the t(9;15) translocation. Five-μm-thick tissue sections were cut for FISH analysis. *SYK* break apart was determined using FISH probe (BAC clones RP11-51G20 and RP11-555F9) and a reference probe, located at 9q22.2. At least 100 nuclei were evaluated per sample using a fluorescence microscope (Olympus BX51; Olympus Optical). *PML* FISH break-apart assay (locus 15q24.1; 74287013-74340155) was performed in the Cytogenetics Laboratory at WCM/NYP following standard operating procedures (Empire Genomics, Williamsville, NY). Cells were isolated by the Ficoll-Paque method from peripheral blood and grown for 3 days in culture media supplemented with 50 µg/mL Pokeweed mitogen (L8777, Sigma-Aldrich). Patient cells were cultured in Colcemide (10295892001, Roche) at 0.1 µg/mL for 1 h.

### Sorting of subpopulations and RNA/DNA extraction

Cells were isolated by the Ficoll-Paque method from peripheral blood (PB) and bone marrow (BM) aspirates. Cells were labeled with anti-human CD45 APC-H7 (BD Biosciences, clone 2D1, cat. 560178), anti-human CD3 PE (BioLegend, clone SK7, cat. 344806) and anti-human CD34 APC (eBioscience, Clone 4H11, cat. 17-0349-42). Cells were washed and resuspended in 4′,6-diamidino-2-phenylindole (DAPI) to exclude dead cells. Populations were sorted using a BD FACSAria-II (BD Biosciences). Total RNA and genomic DNA were extracted from bulk samples and sorted cells with AllPrep DNA/RNA Mini Kit (Qiagen, cat. 80204).

### Reverse-transcriptase polymerase chain reaction (RT-PCR) and PCR

qRT-PCR for PML-SYK and SYK RNA expression was performed using ABL1 as a reference gene to normalize mRNA expression in AML37 and/or normal peripheral blood mononuclear cells with a KAPA SYBR reagent (Kapa Biosystems, KK4601). The sequences of primers are as follows:

PML-SYK forward: GCAGCTGTATCCAAGAAAGCC,

PML-SYK reverse: CCAGGTAAACCTCCTTGGGC,

SYK forward: TGCACTATCGCATCGACAAAG,

SYK reverse: TTGACATGGGACAGTAAGAACT,

ABL1 forward: TGGAGATAACACTCTAAGCATAACTAAAGGT,

ABL1 reverse: GATGTAGTTGCTTGGGACCCA.

The two-step PCR method was performed according to the following protocol: enzyme activation at 95 °C, 20 s, 40 cycles of denaturing step at 95 °C for 2 s and annealing/extension step at 60 °C for 30 s. The product size was confirmed on a 1.5% agarose/TAE gel.

For relative-quantitation of SRY gene (Y chromosome) and GPR34 gene (X chromosome) expression, real-time PCR was performed with sets of primers and TaqMan probe (ThermoFisher Scientific) and TaqMan Gene Expression Master mix (ThermoFisher Scientific cat. 4369016) by using QuantStudio 12 K (ThermoFisher Scientific). Beta-actin (ACTB) gene was used as a reference gene. Primers and TaqMan probe; SRY_Hs00976796_s1 FAM/MGB-NFQ, GPR34_ Hs00271105_s1, VIC/MGB-NFQ and ACTB_Hs01060665_g1 FAM/MGB-NFQ. The RT-PCR method was performed with according to the following protocol: UDG decontamination at 50 °C for 2 min, enzyme activation at 95 °C for 10 min, 35–40 cycles of denaturing step at 95 °C for 15 sec and annealing/extension step at 60 °C for 1 min. The product size was confirmed on a 1.5–4% agarose/TAE or TBE gel.

### PU-FITC-binding assay

PU-FITC-binding assay was performed as described before^1,8^. Evaluation for epichaperome abundance was performed on samples obtained prior treatment with PU-H71, except where indicated (i.e., Fig. 3e, f.) Briefly, isolated cells were incubated with PU-FITC that binds to epichaperome, or FITC9 (FITC control that does not bind to the epichaperome) at 37 °C for 4 h. After washing out PU-FITC or FITC9, cells were labeled with anti-CD45 APC-H7, anti-CD33 PerCP/Cy5.5 (BioLegend, clone WM53, cat. 303414) anti-CD3 PE, and anti-CD34 APC to distinguish subpopulations. Intensity of FITC of each subpopulation was measured by flow cytometry on BD LSRFortessa (BD Biosciences). Data was analyzed using FlowJo software (BD). The epichaperome abundance was evaluated by calculating relative intensity of FITC after correcting the background from FITC9 of each subpopulation to that on T-lymphocytes (CD45^high^CD3 + ) harboring low abundance of epichaperome (MFI of target population/MFI of T-lymphocytes)^8^.

### Cell signaling assay with primary cells

Samples were collected either in Transfix tubes (MBL International) or EDTA tubes. Cells from EDTA tubes were isolated by Ficoll-Paque or RBC lysis. Cells were then fixed by incubating in Fixation Buffer (BD Cytofix,) at 4 °C for 20 min. Transfix samples were processed as per the manufacturer’s instructions. Fixed cells were labeled with cell surface markers, anti-CD45 APC-H7, anti-CD33 PerCP/Cy5.5, anti-CD123 BV650 (BioLegend, clone 6H6, cat. 306020), anti-CD38 PE/Cy7(BioLegend, clone HIT2, cat. 303516), anti-CD34 PE-CF594 (BD Biosciences, clone 581, cat. 562383), and anti-CD3 BV605 (BioLegend, clone SK7, cat. 344836). Subsequently, cells were permeabilized by incubating in Perm/Wash buffer (BD Biosciences) at 4 °C for 30 min. After washing, cells were labeled with anti-phospho-STAT5 AlexaFluor 647 (Y694), anti-phospho-ERK1/2 AlexaFluor 488 (T202/Y204), phospho-S6 V450 (S235/S236) and anti-phospho-SYK PE (Y525/526,) at 4 °C for 15 min. Flow cytometry evaluation was performed on BD LSRFortessa. Data were analyzed using FlowJo software.

### Viability assay

Cells were seeded at 200,000 cells in 200 μL of serum-free culture medium with cytokines (rh FLT3L, rh SCF, rh IL-3 and rh IL6)^6^. Cells were treated at a final concentration of 0.05–1 μM of PU-H71 for 72 h. Cell viability was measured by with 7-AAD (ThermoFisher Scientific, cat. A1310) for dead cells and YO-PRO-I Iodide (ThermoFisher Scientific, cat. Y3603) for apoptotic cells. Flow cytometry evaluation was performed on BD LSRFortessa. Data were analyzed using FlowJo software.

### Colony-forming assays

Baseline PB cells were treated with 100 nM, 500 nM or 1 μM of PU-H71 for 48 h were seeded in duplicate at 25,000 cells per well in 1 mL of methylcellulose-based medium containing rh SCF, rh GM-CSF, rh IL-3, and rh EPO (MethoCult^TM^ Express, STEMCELL TECHNOLOGIES, cat 04437). Colony numbers were counted on day 7.

BM cells at baseline were plated as above and evaluated at day 9 as a reflect of high cell proliferation. BM cells from day 43 were seeded in duplicate at 10,000 cells per 1 mL of MethoCult^TM^ Express and evaluated at day 16.

### Immunophenotyping of T lymphocytes and monocytes

The subsets of T lymphocytes^14,15^ and monocytes^12,13^ at baseline and at following timepoints during PU-H71 treatment were monitored by flow cytometry. Peripheral blood (PB) cells from healthy donor was used as a control. Cells were isolated by the Ficoll-Paque method from PB and bone marrow (BM) aspirates. Cells were labeled with anti-CD45 APC-H7, anti-CD33 BV650 (BD Biosciences, clone WM53, cat. 303430), anti-CD56 AlexaFluor700 (BioLegend, clone HCD56, cat. 318316), anti-CD3 BV711 (BioLegend, clone SK7, cat. 344838), anti-CD4 PE-Cy5 (BioLegend, clone OKT4, cat. 317412), anti-CD8 BV605 (BioLegend, clone SK1, cat. 344742), anti-CD19 PerCP/Cy5.5 (BioLegend, clone HIB19, cat. 302230), anti-CD14 PE (BD Biosciences, clone M5E2, cat. 555398), anti-CD16 BV785 (BioLegend, clone 3G8, cat. 302046), anti-CD45RA PE-Cy7 (BioLegend, clone HI100, cat. 304126) and anti-CCR7 Alexa Fluor 647 (BD Biosciences, clone 150503, cat. 560816). After washing, flow cytometry evaluation was performed on BD LSRFortessa. Data was analyzed using FlowJo software.

### Epichaperome analysis by the NanoPro capillary-based immunoassay platform

Epichaperome abundance in CD34 + blasts in WCM254 at baseline and on day 5 after starting treatment was measured by using a capillary-based platform that combines isoelectric focusing (IEF) with immunoblotting capabilities as described before^1^. Cells were lysed in 20 mM HEPES pH 7.5, 50 mM KCL, 5 mM MgCl_2_, 0.01% NP40, 20 mM Na_2_MoO_4_ buffer containing protease and phosphatase inhibitors. Total protein assay to detect epichaperome was performed on an automated system, NanoPro 1000 Simple Western (ProteinSimple), for charge-based separation. Samples were loaded into capillaries (ProteinSimple) and separated based on their isoelectric points. Immobilization was performed by UV-light embedded, followed by labeling with anti-HSP90β (SMC-107A, StressMarq Biosciences) and subsequently with HRP-conjugated anti-Mouse IgG (1030-5, SouthernBiotech) or with HRP-conjugated anti-Rabbit IgG (4010-05, SouthernBiotech). Protein signals were quantified by chemiluminescence using SuperSignal West Dura Extended Duration Substrate (Thermo Scientific), and digital imaging and associated software (Compass) in the Simple Western system, resulting in a gel-like representation of the chromatogram.

### Ethics declarations

All experimental procedures were carried out in accordance with approved guidelines and were approved by the Institutional Review Board (IRB) at Weill Cornell Medicine. Patients in the study signed informed written consent under an IRB-approved protocol (IRB #1305013903).

### Reporting summary

Further information on research design is available in the Nature Research Reporting Summary linked to this article.

## Supplementary information

Supplementary Information

Reporting Summary

## Data Availability

The data generated and analyzed during this study are described in the following figshare data record: 10.6084/m9.figshare.13366430^25^. All data are shared openly. The RNA-sequencing and whole-exome sequencing data are shared in the European Genotype-phenotype Archive under accession https://identifiers.org/ega.study:EGAS00001004992^26^. All other data are shared as part of the figshare data record. A list of which datafiles underlie which figures in the manuscript is also included in Excel.xlsx format in the file “sugita_datafiles_underlying_figures_lookup.xlsx.”

## Code availability

Data was generated using publicly available tools described in methods section.
