# Supplementary material for: Targeting the epichaperome as an effective precision medicine approach in a novel PML-SYK fusion acute myeloid leukemia
Source: NPJ Precis Oncol. 2021 May 26;5:44. doi: 10.1038/s41698-021-00183-2 (PMC8155064; doi:10.1038/s41698-021-00183-2)

Supplementary Table 1.

ID	Sample	Initial Diagnosis	SNV and CNV detected by WES	Gene fusions detected by RNA-seq
**WCM254**	Bone marrow	MPD, unclassified	ASXL p.L775*<br>IDH1 p.D137Y<br>PPM1D p.L484*	PML--SYK
**WCM319**	Bone marrow	AML	RB1 focal amplification<br>SUZ12 focal deletion<br>NF1 focal deletion	NP
**WCM397**	Bone marrow	Myeloid hyperplasia	TCHH p.D1624E<br>FDX1 focal deletion<br>TLK1 focal amplification	AKR1C1--AKR1E2<br>EDF1--RABL6<br>PIM3--SCO2
**WCM401**	Bone marrow	AML	FOXP1 focal deletion<br>RIPK4 p.R558C<br>GPR39 p.V294I	NP
**WCM586**	Bone marrow	Chronic Eosinophilic Leukemia	JAK2 focal deletion<br>SLC45A1 p.F272L<br>PHYH focal deletion	NP
**WCM1293**	Bone marrow	Atypical CML	RANBP17 focal amplification<br>EPPK1 p.A1954T<br>PLAGL1 p.L298P	NP
**WCM1447**	Skin	Langerhans Cell Sarcoma	KRAS p.Q61R<br>FOXA1 p.S395F<br>BCL6 p.S532X	ND

Supplementary Table 1. Initial diagnoses of patients with myeloid neoplasia enrolled in the NGS-based clinical study at time of manuscript submission. Whole-exome sequencing (WES) and RNA-seq elucidated molecular alterations that included a novel PML-SYK gene fusion in index patient WCM254. (SNV=Single nucleotide variant, CNA=copy number alteration, NP=Not performed, ND=No fusion detected).

Supplementary figure 1

a Fluorescence in situ hybridization using the LSI PML-RARA probes

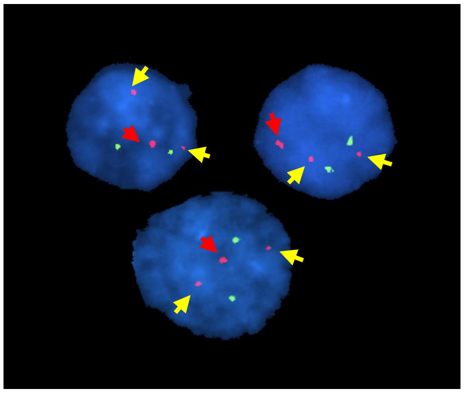

The yellow arrows indicate the split PML signals.
Red arrows indicate normal PML allele.

b Karyotype showing the the involvement of PML gene in a translocation

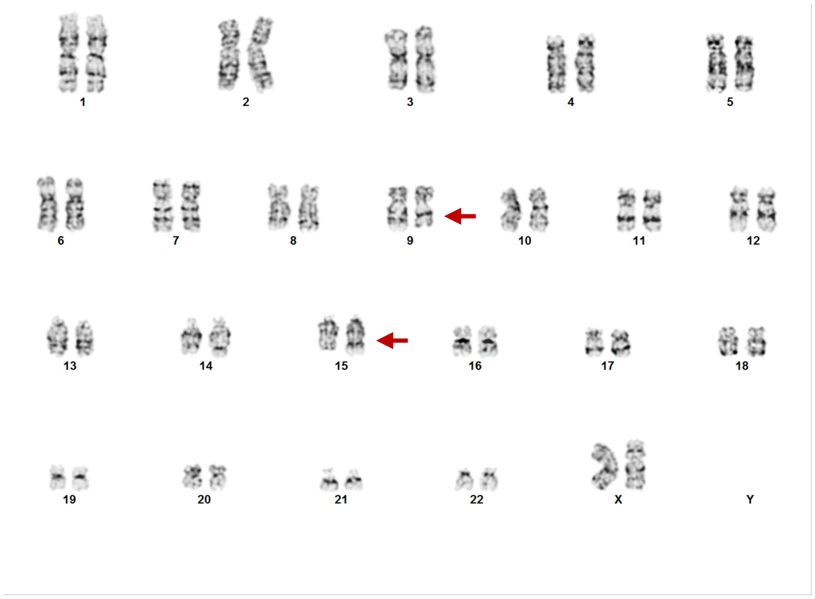

c

d

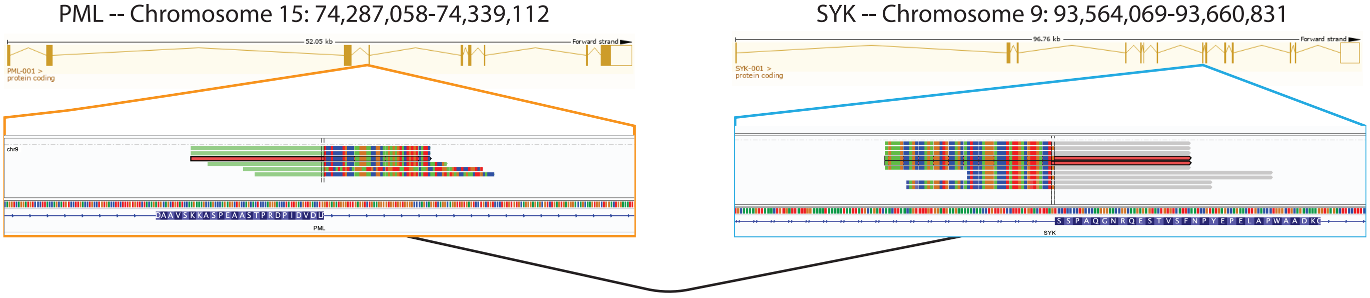

e

ENSP00000268058-ENSP00000364907 length=748, kD: 84.9327011, transcripts: ENST00000268058-ENST00000375754, genes: PML-SYK, effect: in-frame

361 | 395 | 419 | 449 | 480

[...] QEEPQSLQAAVRTDGFDEFKVRLQDLSSCITQGKDAAVSKKASPEAASTPRDPIDVDLSSPAQGNRQESTVSFNPYEPELAPWAADKGPQREALPMDTEVYESPYADPEEIRPKEVYLDR [...]

f

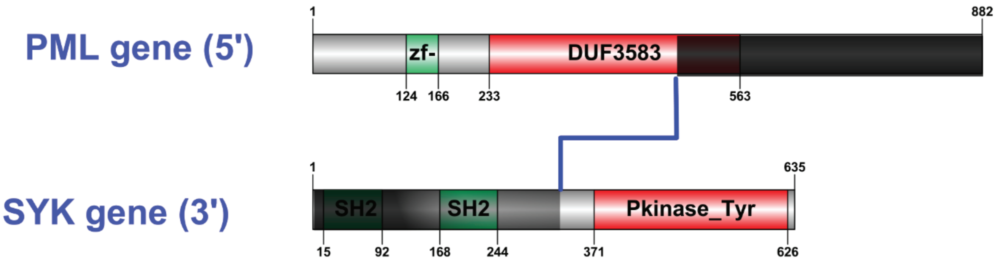

g

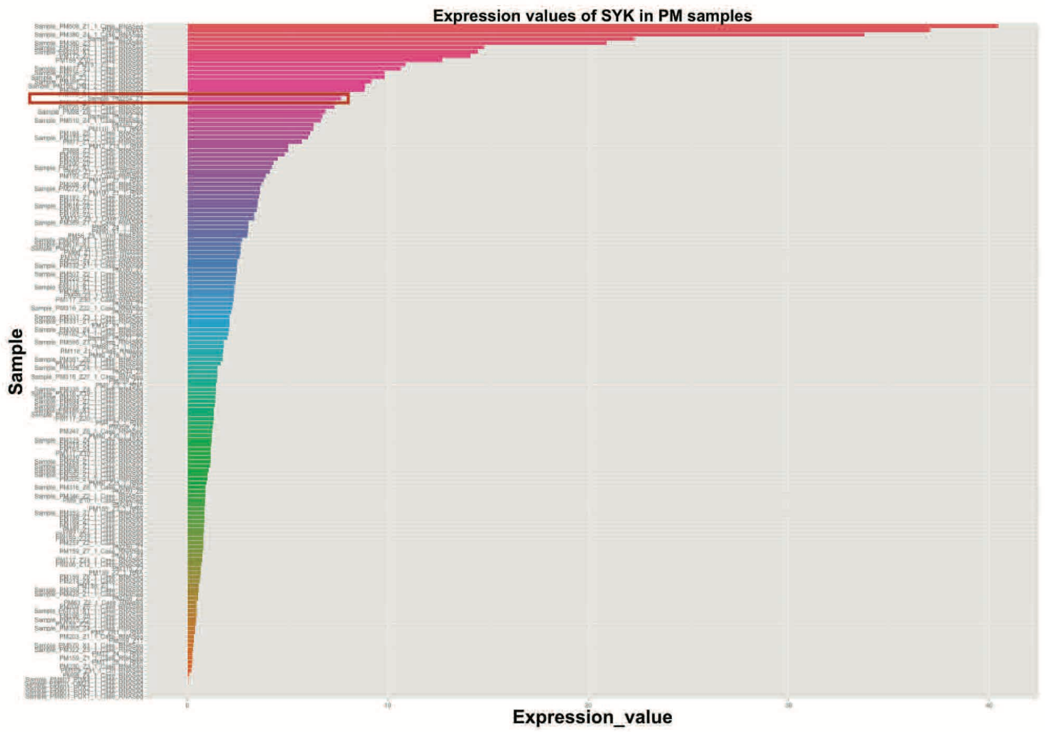

h

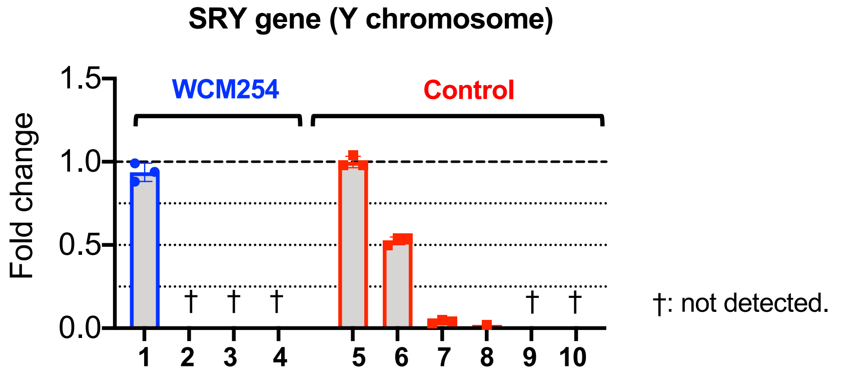

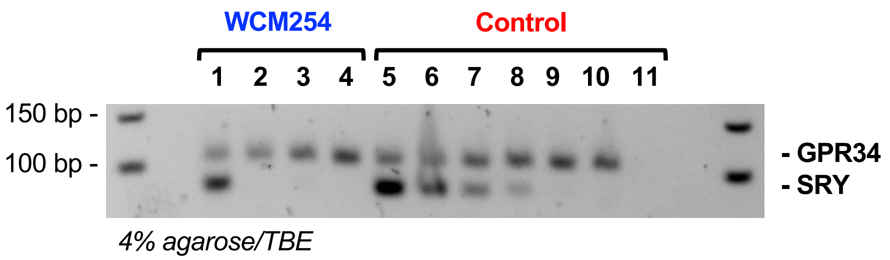

WCM254
1 WCM254 lymphocytes
2 WCM254 CD34+blasts
3 WCM254 monocytes
4 WCM254 granulocytes

Controls
5 M 100%
6 M 50%, : F 50%
7 M 5%, : F 95%
8 M 1%, : F 99%
9 M 0.1%, : F 99.9%
10 F 100%
11 NTC

*M; MV4;11 (male), F; HL60 (female)
GPR34 gene: X chromosome
SRY gene: Y chromosome

Supplementary Figure 1. Precision medicine approaches identified novel PML-SYK translocation with epichaperome dependency. **a.** Fluorescence in situ hybridization using the LSI PML-RARA probes. PML is labeled with spectrum orange and RARA is labeled with spectrum green. The yellow arrows indicate the split PML signals. Red arrows indicate normal PML allele. **b.** Karyogram of WCM254 and a karyotype 46,XX,t(9;15)(q22;q22). The arrows indicate the abnormal chromosome 9 and 15. **c.** Genomic representation of PML (left) and SYK (right). **d.** Zoomed in on the exonic areas involved in the fusion: exon 4 of PML (left) and exon 9 of SYK (right). Reads from the index patient data that support the fusion transcripts are here represented via the colored soft-clipped based for the Integrative Genome Viewer (IGV). **e.** Resulting protein sequence near the fusion junction. The numbers above the sequence corresponds to the amino acid positionThe colored amino acids correspond to those highlighted in panel *d.* **f.** Schematic of the full protein sequence highlighting the protein domains. Shaded areas are not included in the fusion protein. **g.** Overexpression of SYK when RNA-seq data from the index case WCM254 was compared with gene expression in EIPM pan-cancer cohort. **h.** RT-PCR to distinguish donor (male) from recipient (female) cells; SRY gene (Y chromosome) and GPR34 gene (X chromosome) were evaluated in the indicated sorted subsets of WCM254. As controls, MV4;11 (male AML cell line) and HL60 (female AML cell line) were used at the indicated ratios (lane 5-10), no template control (NTC) was also included (lane 11). (Top) Change of SRY gene expression relative to MV4;11 in each subset of WCM254 is shown. *(bottom)* Electrophoresis of PCR products showing a band sized 84 bp for the SRY (Y chromosome) and 109 bp for the GPR34 (X chromosome).

Supplementary figure 2

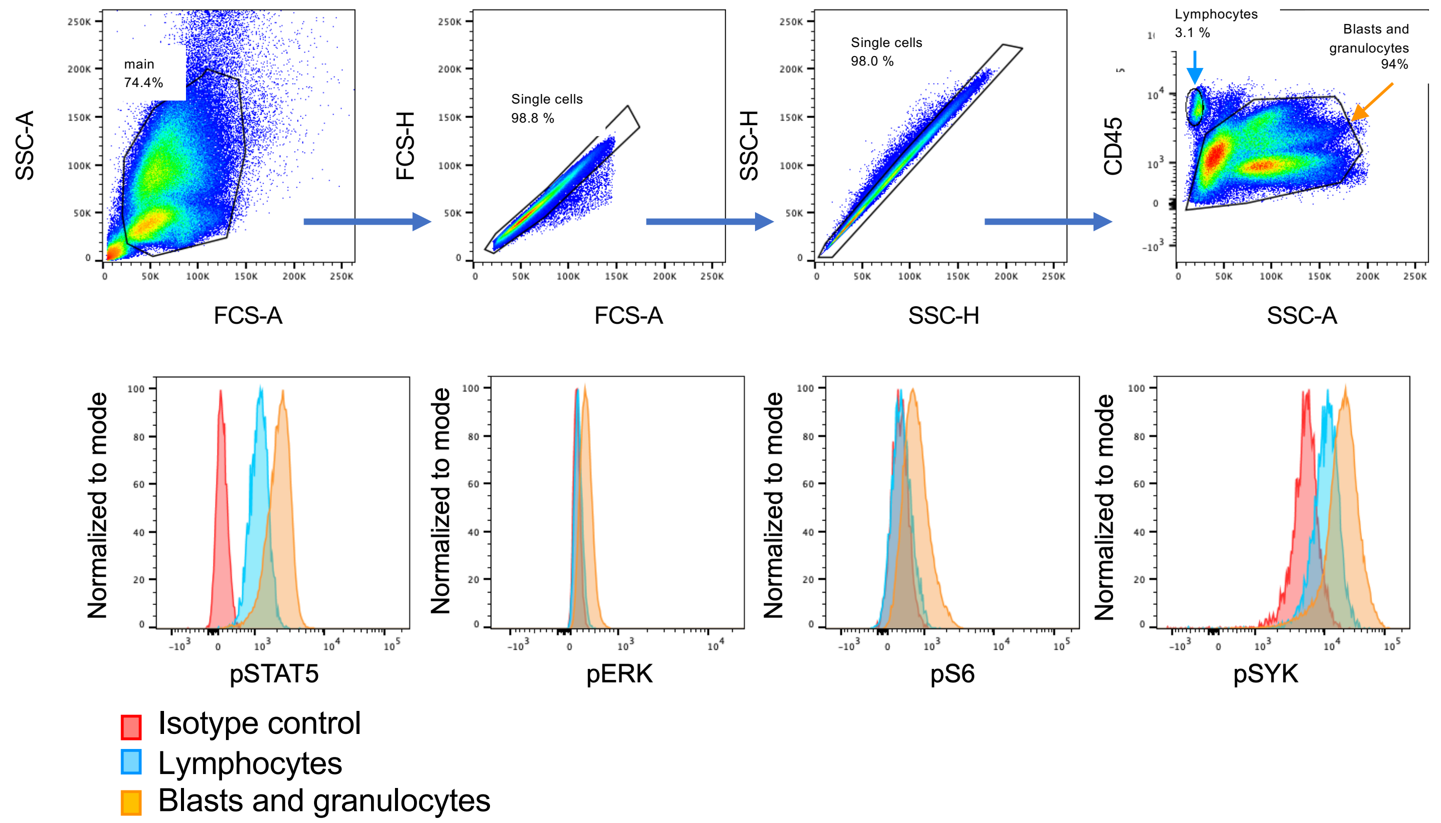

Supplementary Figure 2. Evaluation of signaling pathways in cells from the WCM254 patient. (Top) Flow cytometry gating strategy, debris were first gated out, followed by gating for single cells. Lymphocytes, blasts and granulocytes were gated based on SSC vs CD45. (Bottom) Histograms for p-STAT5 (Y694), p-ERK1/2 (T202/Y204), p-S6 (S235/S236) and p-SYK (Y525/526) of each of the populations. Representative example from PB sample at baseline.

Supplementary figure 3

a

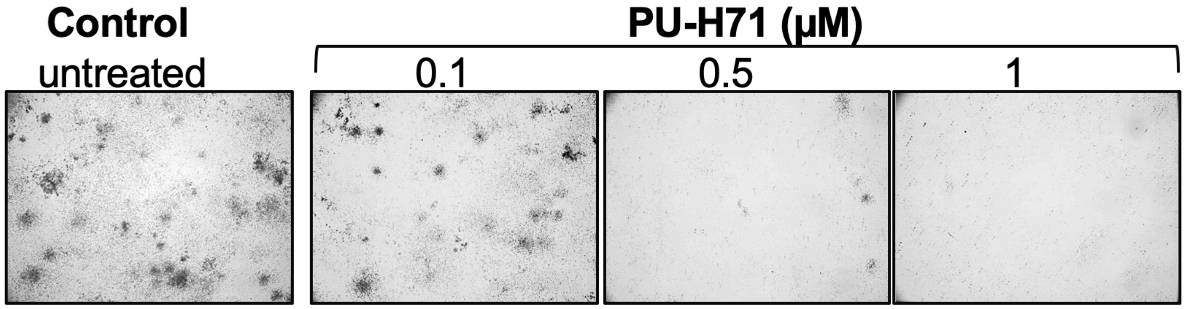

b

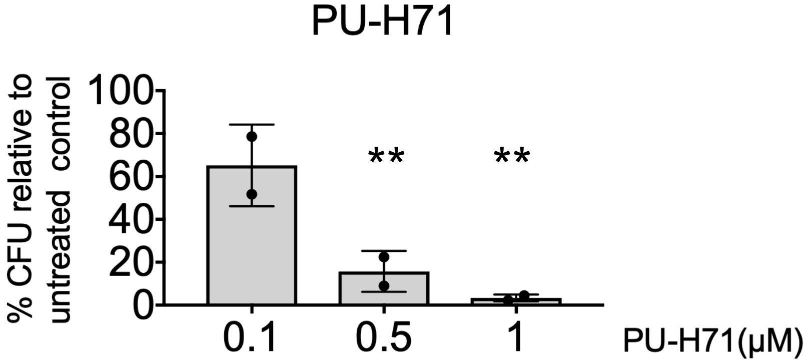

Supplementary Figure 3. PU-H71 *ex vivo* treatment inhibited colony formation of WCM254. a. Representative images of formed colonies after plating with cells from peripheral blood (PB) WCM254 baseline sample treated with the indicated concentrations of PU-H71 for 48h. **b.** Percent CFU relative to control in colony-forming assays of WCM254 cells. Each symbol represents one well in replicates and bar presents the mean with the SD. ** *p<0.01,* one-way ANOVA

Supplementary figure 4

WMC254 BM Baseline

WMC254 BM Day 43

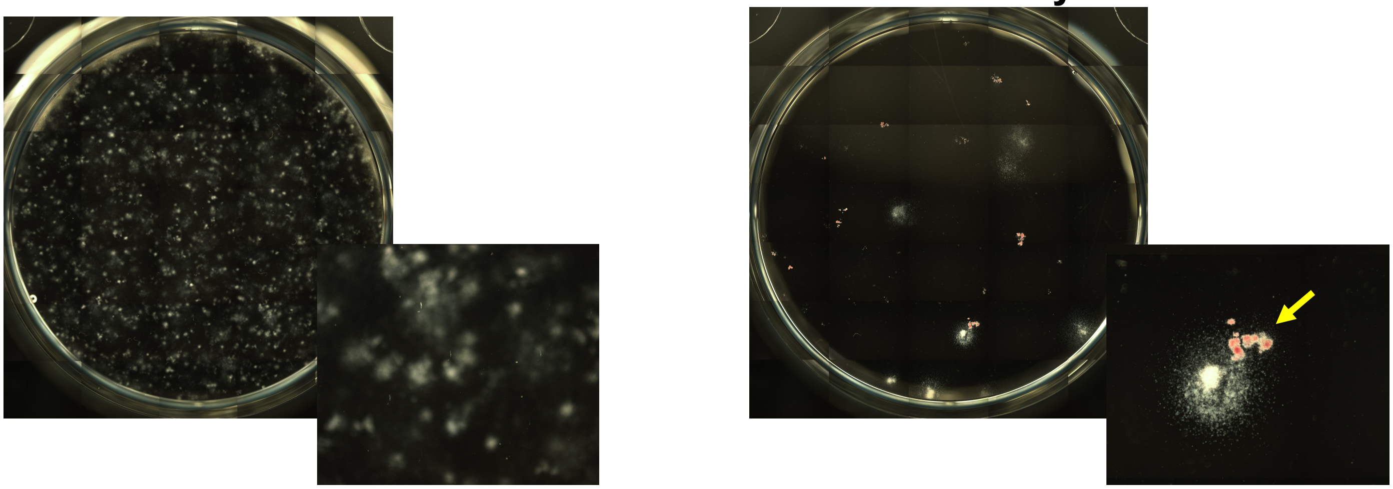

Supplementary Figure 4. PU-H71 *in vivo* treatment inhibited colony formation of WCM254. Representative pictures of CFU assays performed to assesses WMC254 bone marrow (BM) samples from the indicated timepoints. Inset shows a closer section of the plate. Arrow indicates erythroid colony.

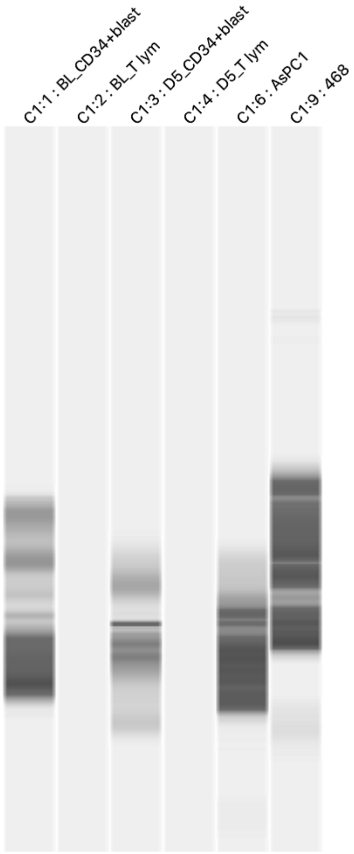

Full image for 2e Capillary isoelectric focusing electrophoresis using anti-HSP90β showing long-lived multimeric HSP90 species (a biochemical signature of epichaperomes) in sorted CD34+ baseline (BL) blasts that decrease by day 5 of treatment (D5). Negative control, ASPC1 (epichaperome +) homogenate; positive control, MDA-MB-468 homogenate (epichaperome-). NanoPro profiles for each of the lanes are shown below.

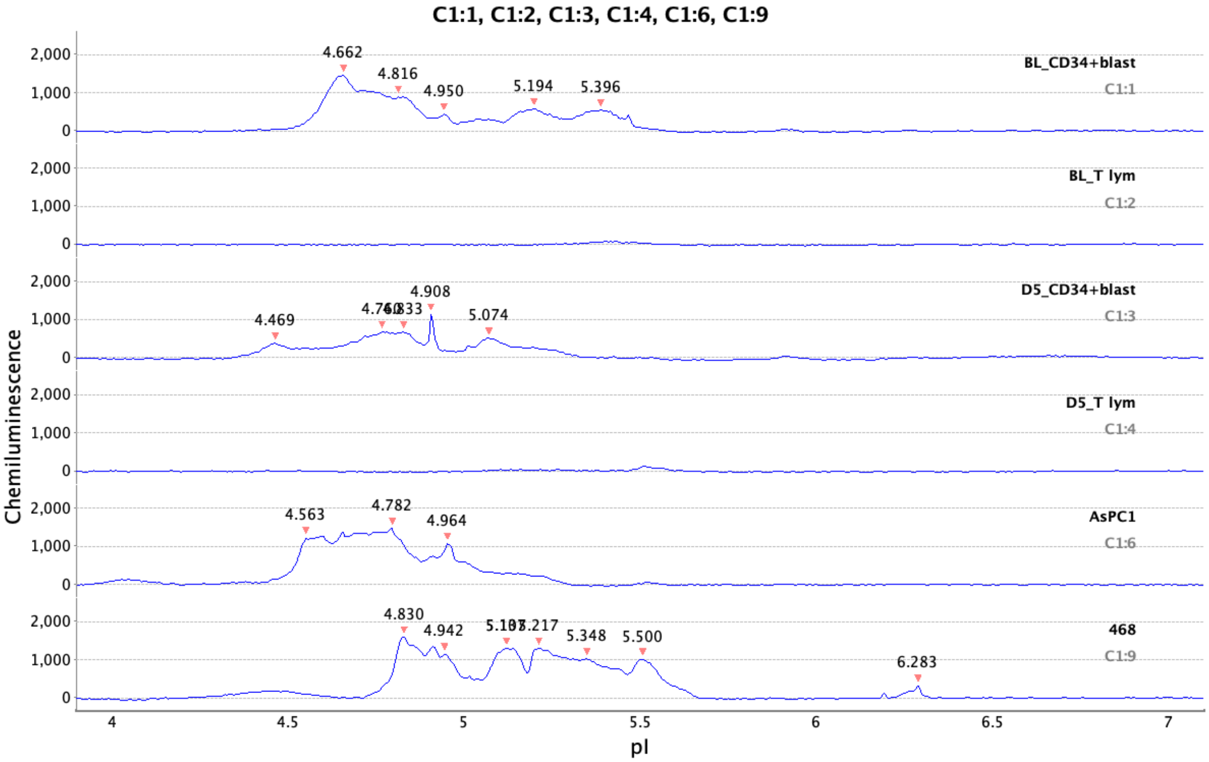

Supplement: Supplementary file 1 — Supplementary Information [file 41698_2021_183_MOESM1_ESM.pdf]
