# Supplementary material for: Targeting the epichaperome as an effective precision medicine approach in a novel PML-SYK fusion acute myeloid leukemia
Source: NPJ Precis Oncol. 2021 May 26;5:44. doi: 10.1038/s41698-021-00183-2 (PMC8155064; doi:10.1038/s41698-021-00183-2)
Supplement: Supplementary file 2 — Reporting Summary [file 41698_2021_183_MOESM2_ESM.pdf]

nature research

Corresponding author(s): Gail J. Roboz, Juan Miguel Mosquera, M.D. Monica L. Guzman, Ph.D.

Last updated by author(s): 2020-10-16

Reporting Summary

Nature Research wishes to improve the reproducibility of the work that we publish. This form provides structure for consistency and transparency in reporting. For further information on Nature Research policies, see our Editorial Policies and the Editorial Policy Checklist.

Please do not complete any field with "not applicable" or n/a. Refer to the help text for what text to use if an item is not relevant to your study. For final submission: please carefully check your responses for accuracy; you will not be able to make changes later.

Statistics

For all statistical analyses, confirm that the following items are present in the figure legend, table legend, main text, or Methods section.

n/a	Confirmed	
☐	☒	The exact sample size (*n*) for each experimental group/condition, given as a discrete number and unit of measurement
☐	☒	A statement on whether measurements were taken from distinct samples or whether the same sample was measured repeatedly
☐	☒	The statistical test(s) used AND whether they are one- or two-sided<br>*Only common tests should be described solely by name; describe more complex techniques in the Methods section.*
☐	☒	A description of all covariates tested
☐	☒	A description of any assumptions or corrections, such as tests of normality and adjustment for multiple comparisons
☐	☒	A full description of the statistical parameters including central tendency (e.g. means) or other basic estimates (e.g. regression coefficient) AND variation (e.g. standard deviation) or associated estimates of uncertainty (e.g. confidence intervals)
☐	☒	For null hypothesis testing, the test statistic (e.g. F, t, r) with confidence intervals, effect sizes, degrees of freedom and P value noted<br>*Give P values as exact values whenever suitable.*
☒	☐	For Bayesian analysis, information on the choice of priors and Markov chain Monte Carlo settings
☒	☐	For hierarchical and complex designs, identification of the appropriate level for tests and full reporting of outcomes
☒	☐	Estimates of effect sizes (e.g. Cohen's d, Pearson's r), indicating how they were calculated

Our web collection on statistics for biologists contains articles on many of the points above.

Software and code

Policy information about availability of computer code

Data collection: *Provide a description of all commercial, open source and custom code used to collect the data in this study, specifying the version used OR state that no software was used.*

Data analysis: *Provide a description of all commercial, open source and custom code used to analyse the data in this study, specifying the version used OR state that no software was used.*

For manuscripts utilizing custom algorithms or software that are central to the research but not yet described in published literature, software must be made available to editors and reviewers. We strongly encourage code deposition in a community repository (e.g. GitHub). See the Nature Research guidelines for submitting code & software for further information.

Data

Policy information about availability of data

All manuscripts must include a data availability statement. This statement should provide the following information, where applicable:
- Accession codes, unique identifiers, or web links for publicly available datasets
- A list of figures that have associated raw data
- A description of any restrictions on data availability

The data that support the findings of this study are available from the corresponding authors upon reasonable request.

April 2020

Field-specific reporting

Please select the one below that is the best fit for your research. If you are not sure, read the appropriate sections before making your selection.

- [x] Life sciences
- [ ] Behavioural & social sciences
- [ ] Ecological, evolutionary & environmental sciences

For a reference copy of the document with all sections, see nature.com/documents/nr-reporting-summary-flat.pdf

Life sciences study design

All studies must disclose on these points even when the disclosure is negative.

Sample size	*This is a case report presenting the data of a single patient*
Data exclusions	*There were no data exclusions*
Replication	When material available data was replicated, figures show the replicates as single points in the plots presented.
Randomization	*This is not relevant to our study as it is a case report.*
Blinding	*This is not relevant to our study as it is a case report.*

Behavioural & social sciences study design

All studies must disclose on these points even when the disclosure is negative.

Study description	*N/A*
Research sample	*N/A*
Sampling strategy	*N/A*
Data collection	*N/A*
Timing	*N/A*
Data exclusions	*N/A*
Non-participation	*N/A*
Randomization	*N/A*

Ecological, evolutionary & environmental sciences study design

All studies must disclose on these points even when the disclosure is negative.

Study description	*N/A*
Research sample	*N/A*

Research sample — *Monument), and provide a rationale for the sample choice. When relevant, describe the organism taxa, source, sex, age range and any manipulations. State what population the sample is meant to represent when applicable. For studies involving existing datasets, describe the data and its source.*

Sampling strategy — *Note the sampling procedure. Describe the statistical methods that were used to predetermine sample size OR if no sample-size calculation was performed, describe how sample sizes were chosen and provide a rationale for why these sample sizes are sufficient.*

Data collection — *Describe the data collection procedure, including who recorded the data and how.*

Timing and spatial scale — *Indicate the start and stop dates of data collection, noting the frequency and periodicity of sampling and providing a rationale for these choices. If there is a gap between collection periods, state the dates for each sample cohort. Specify the spatial scale from which the data are taken*

Data exclusions — *If no data were excluded from the analyses, state so OR if data were excluded, describe the exclusions and the rationale behind them, indicating whether exclusion criteria were pre-established.*

Reproducibility — *Describe the measures taken to verify the reproducibility of experimental findings. For each experiment, note whether any attempts to repeat the experiment failed OR state that all attempts to repeat the experiment were successful.*

Randomization — *Describe how samples/organisms/participants were allocated into groups. If allocation was not random, describe how covariates were controlled. If this is not relevant to your study, explain why.*

Blinding — *Describe the extent of blinding used during data acquisition and analysis. If blinding was not possible, describe why OR explain why blinding was not relevant to your study.*

Did the study involve field work? [ ] Yes [ ] No

Field work, collection and transport

Field conditions — *N/A*

Location — *N/A*

Access & import/export — *N/A*

Disturbance — *N/A*

Reporting for specific materials, systems and methods

We require information from authors about some types of materials, experimental systems and methods used in many studies. Here, indicate whether each material, system or method listed is relevant to your study. If you are not sure if a list item applies to your research, read the appropriate section before selecting a response.

Materials & experimental systems

n/a	Involved in the study	
[ ]	[x]	Antibodies
[ ]	[x]	Eukaryotic cell lines
[x]	[ ]	Palaeontology and archaeology
[x]	[ ]	Animals and other organisms
[ ]	[x]	Human research participants
[x]	[ ]	Clinical data
[x]	[ ]	Dual use research of concern

Methods

n/a	Involved in the study	
[x]	[ ]	ChIP-seq
[ ]	[x]	Flow cytometry
[x]	[ ]	MRI-based neuroimaging

Antibodies

Antibodies used — ***All described in methods:*** *anti-CD45 APC-H7, anti-CD33 BV650 (BD Biosciences, clone WM53, cat. 303430), anti-CD56 AlexaFluor700 (BioLegend, clone HCD56, cat. 318316), anti-CD3 BV711 (BioLegend, clone SK7, cat. 344838), anti-CD4 PE-Cy5 (BioLegend, clone OKT4, cat. 317412), anti-CD8 BV605 (BioLegend, clone SK1, cat. 344742), anti-CD19 PerCP/Cy5.5 (BioLegend, clone HIB19, cat. 302230), anti-CD14 PE (BD Biosciences, clone M5E2, cat. 555398), anti-CD16 BV785 (BioLegend, clone 3G8, cat. 302046), anti-CD45RA PE-Cy7 (BioLegend, clone HI100, cat. 304126) and anti-CCR7 Alexa Fluor 647 (BD Biosciences, clone 150503, cat. 560816). anti-HSP90β (SMC-107A, StressMarq Biosciences) and subsequently with HRP-conjugated anti-Mouse IgG (1030-5, SouthernBiotech) or with HRP-conjugated anti-Rabbit IgG (4010-05, SouthernBiotech).*

Validation — *All antibodies were used with positive and negative controls, as well as with FMO controls in the case of flow cytometry. All antibodies were obtained from commercially available sources.*

Eukaryotic cell lines

Policy information about cell lines

Cell line source(s) — *MV4;11 cell line was obtained from the ATCC (Manassas, VA, USA)*

Authentication | *Cell lines are sent for authentication when expanded and stored in large batches for experiments.*

Mycoplasma contamination | *We routinely check for mycoplasma using two methods PCR based and colorimetric based*

Commonly misidentified lines (See ICLAC register) | *NA*

Palaeontology and Archaeology

Specimen provenance | NA

Specimen deposition | NA

Dating methods | NA

☐ Tick this box to confirm that the raw and calibrated dates are available in the paper or in Supplementary Information.

Ethics oversight | NA

Note that full information on the approval of the study protocol must also be provided in the manuscript.

Animals and other organisms

Policy information about studies involving animals; ARRIVE guidelines recommended for reporting animal research

Laboratory animals | NA

Wild animals | NA

Field-collected samples | NA

Ethics oversight | NA

Note that full information on the approval of the study protocol must also be provided in the manuscript.

Human research participants

Policy information about studies involving human research participants

Population characteristics | *Single patient case report*

Recruitment | *Single patient case report*

Ethics oversight | *WCM IRB*

Note that full information on the approval of the study protocol must also be provided in the manuscript.

Clinical data

Policy information about clinical studies

All manuscripts should comply with the ICMJE guidelines for publication of clinical research and a completed CONSORT checklist must be included with all submissions.

Clinical trial registration | *NA*

Study protocol | *NA*

Data collection | *NA*

Outcomes | *NA*

Dual use research of concern

Policy information about dual use research of concern

Hazards

Could the accidental, deliberate or reckless misuse of agents or technologies generated in the work, or the application of information presented in the manuscript, pose a threat to:

No	Yes	
☐	☐	Public health
☐	☐	National security
☐	☐	Crops and/or livestock
☐	☐	Ecosystems
☐	☐	Any other significant area

Experiments of concern

Does the work involve any of these experiments of concern:

No	Yes	
☒	☐	Demonstrate how to render a vaccine ineffective
☒	☐	Confer resistance to therapeutically useful antibiotics or antiviral agents
☒	☐	Enhance the virulence of a pathogen or render a nonpathogen virulent
☒	☐	Increase transmissibility of a pathogen
☒	☐	Alter the host range of a pathogen
☒	☐	Enable evasion of diagnostic/detection modalities
☒	☐	Enable the weaponization of a biological agent or toxin
☒	☐	Any other potentially harmful combination of experiments and agents

ChIP-seq

Data deposition

☐ Confirm that both raw and final processed data have been deposited in a public database such as GEO.

☐ Confirm that you have deposited or provided access to graph files (e.g. BED files) for the called peaks.

Data access links<br>*May remain private before publication.*	*NA.*
Files in database submission	*NA*
Genome browser session<br>(e.g. UCSC)	*NA*

Methodology

Replicates	*NA*
Sequencing depth	*NA*
Antibodies	*NA*
Peak calling parameters	*NA*
Data quality	*NA*
Software	*NA*

Flow Cytometry

Plots

Confirm that:

■ The axis labels state the marker and fluorochrome used (e.g. CD4-FITC).

■ The axis scales are clearly visible. Include numbers along axes only for bottom left plot of group (a 'group' is an analysis of identical markers).

■ All plots are contour plots with outliers or pseudocolor plots.

■ A numerical value for number of cells or percentage (with statistics) is provided.

Methodology

Sample preparation	*Described in the methods page 15-17.*
Instrument	*Described in methods: BD LSR-Fortessa*
Software	*Data is collected using BD FACS DiVa v8 0 1 and analyzed using FlowJo software 10 7 1*
Cell population abundance	*For sorts, lymphocyte abundance was 159,000- 655,000; blasts 65,000 – 4,600,000; monocytes 33,000 – 168,000; and granulocytes 81,000 – 1,200,000. Purities were always confirmed after sort and were above 95%*
Gating strategy	*All gating strategies are presented in the manuscript*

■ Tick this box to confirm that a figure exemplifying the gating strategy is provided in the Supplementary Information.

Magnetic resonance imaging

Experimental design

Design type	*NA*
Design specifications	*NA*
Behavioral performance measures	*NA*

Acquisition

Imaging type(s)	*NA*
Field strength	*NA*
Sequence & imaging parameters	*NA*
Area of acquisition	*NA*

Diffusion MRI ☐ Used ☐ Not used

Preprocessing

Preprocessing software	*NA*
Normalization	*NA*
Normalization template	*NA*
Noise and artifact removal	*NA*

Volume censoring | *NA*

Statistical modeling & inference

Model type and settings | *NA*

Effect(s) tested | *NA*

Specify type of analysis: ☐ Whole brain ☐ ROI-based ☐ Both

Statistic type for inference
(See Eklund et al. 2016) | *NA*

Correction | *NA*

Models & analysis

n/a	Involved in the study
☐	☐ Functional and/or effective connectivity
☐	☐ Graph analysis
☐	☐ Multivariate modeling or predictive analysis

Functional and/or effective connectivity | *NA*

Graph analysis | *NA*

Multivariate modeling and predictive analysis | *NA*

nature research | reporting summary
April 2020

[illegible]is checklist template is licensed under a Creative Commons Attribution 4.0 International License, which permits use, sharing, adaptation, distribution and reproduction in any medium or format, as long as you give [illegible]ppropriate credit to the original author(s) and the source, provide a link to the Creative Commons license, and indicate if changes were made. The images or other third party material in this article are included in [illegible]e article's Creative Commons license, unless indicated otherwise in a credit line to the material. If material is not included in the article's Creative Commons license and your intended use is not permitted by [illegible]atutory regulation or exceeds the permitted use, you will need to obtain permission directly from the copyright holder. To view a copy of this license, visit http://creativecommons.org/licenses/by/4.0/
